# The Methyltransferase WBSCR22/Merm1 Enhances Glucocorticoid Receptor Function and Is Regulated in Lung Inflammation and Cancer*[Fn FN2]

**DOI:** 10.1074/jbc.M113.540906

**Published:** 2014-01-31

**Authors:** Maryam Jangani, Toryn M. Poolman, Laura Matthews, Nan Yang, Stuart N. Farrow, Andrew Berry, Neil Hanley, Andrew J. K. Williamson, Anthony D. Whetton, Rachelle Donn, David W. Ray

**Affiliations:** From the ‡ Centre in Endocrinology and Diabetes, Institute of Human Development, and; § the Arthritis Research UK Epidemiology Unit, University of Manchester and Manchester Academic Health Science Centre, Manchester M13 9PT, United Kingdom and; ¶ Stem Cell and Leukaemia Proteomics Laboratory, Faculty Institute for Cancer Sciences, University of Manchester, Manchester Academic Health Science Centre, Manchester M13 9PT, United Kingdom

**Keywords:** Glucocorticoid Receptor, Glucocorticoids, Histone Methylation, Inflammation, Interferon, Lung, Tumor Necrosis Factor (TNF), Ubiquitination

## Abstract

Glucocorticoids (GC) regulate cell fate and immune function. We identified the metastasis-promoting methyltransferase, metastasis-related methyltransferase 1 (WBSCR22/Merm1) as a novel glucocorticoid receptor (GR) regulator relevant to human disease. Merm1 binds the GR co-activator GRIP1 but not GR. Loss of Merm1 impaired both GR transactivation and transrepression by reducing GR recruitment to its binding sites. This was accompanied by loss of GR-dependent H3K4Me3 at a well characterized promoter. Inflammation promotes GC resistance, in part through the actions of TNFα and IFNγ. These cytokines suppressed Merm1 protein expression by driving ubiquitination of two conserved lysine residues. Restoration of Merm1 expression rescued GR transactivation. Cytokine suppression of Merm1 and of GR function was also seen in human lung explants. In addition, striking loss of Merm1 protein was observed in both inflammatory and neoplastic human lung pathologies. In conclusion, Merm1 is a novel regulator of chromatin structure affecting GR recruitment and function, contributing to loss of GC sensitivity in inflammation, with suppressed expression in pulmonary disease.

## Teaser

**Background:** Glucocorticoid (GC) action in inflammation is important but poorly understood.

**Results:** Merm1 regulates glucocorticoid receptor (GR) recruitment to the genome and mediates subsequent histone modification. Proinflammatory cytokine induction of GC resistance is accompanied by Merm1 degradation; restoration of Merm1 expression rescues GC action.

**Conclusion:** Merm1 is a novel GR co-modulator.

**Significance:** Merm1 mediates inflammation regulation of GC action.

## Introduction

Glucocorticoids (GC)^6^ exert diverse effects on multiple cell types and tissues, affecting energy metabolism, cell fate, and differentiated function. Variation in GC sensitivity is evident in several disease states, with resistance occurring in chronic inflammatory diseases including rheumatoid arthritis, asthma, and chronic obstructive pulmonary disease through mechanisms that remain undefined but are thought to result from cytokine action (1–4). In addition, human small cell lung cancer is characterized by GC resistance, which prevents GC induction of cell death both *in vitro* and *in vivo* (5, 6).

The diverse actions of GC are mediated through the ubiquitously expressed glucocorticoid receptor (GR). GR is a nuclear hormone receptor that acts as a ligand-inducible transcription factor interacting with chromatin to regulate gene transcription (7–9). Selection of GR binding sites is dependent on cell type-specific chromatin structure, which regulates accessibility to target DNA, giving rise to cell type-specific GR cistromes (10–13).

Regulation of transcription by GR is mediated by co-modulator proteins, some of which regulate post-translational modification of histone proteins (*e.g.* p160, CARM1, CBP) and some that do not (*e.g.* SWI/SNF) (14, 15). Changes in post-translational modifications on core histone tails, particularly acetylation and methylation, critically affect chromatin structure and gene expression. Much attention has been given to histone acetylation, a transient mark that renders nucleosomal DNA more accessible to protein binding (16). However, histone methylation is also important. In the context of GR-regulated gene transcription the histone arginine methyltransferase CARM1 plays a prominent role (17).

Histone acetylation regulates the charge on the histone tail and relaxes the association with DNA, thereby “opening” chromatin to transcription factor binding. In contrast, histone methylation has no effect on charge but likely serves as a protein recognition surface (18, 19). Histone methylation involves three groups of protein complexes: “writers,” or histone methyltransferases, “erasers,” or histone demethylases, and “readers,” or proteins recruited to the methylated histones. Three families of enzymes result in histone methylation. The SET domain, and DOT1-like proteins both methylate lysine, and the protein arginine *N*-methyltransferase (PRMT) family methylate arginine residues (20). Recent evidence points toward coordinated modification of multiple histone residues at a given locus, an effect regulated by long non-coding RNAs (lncRNA), such as *hotair* (20).

We have identified the WBSCR22/metastasis-related methyltransferase 1 (*Merm1*) gene as an important regulator of GR binding and function and a mediator of cytokine-induced glucocorticoid resistance. *Merm1/wbscr22* is one of 26–28 genes deleted from 7q11.23 in Williams-Beuren syndrome, a developmental disorder with multisystem manifestations, including glucose intolerance and diabetes mellitus (21).

Merm1 is highly conserved through evolution from *Saccharomyces cerevisiae* and *Caenorhabditis elegans* to humans. RNAi-mediated knockdown of the Merm1 ortholog in *C. elegans* is embryonic lethal (22). Based on the Hidden Markov Model of sequence and three-dimensional structural analysis, Merm1 has been categorized into the seven-β-strand family of methyltransferases, which includes the arginine methyltransferase family (including CARM1, PRMT1) and the non-SET domain lysine methyltransferases (including DOT1L) (23). Although there is strong evidence implicating the arginine methyltransferases, and the SET domain methyltransferases in regulating access of nuclear receptors to target sites and also in mediating their effects on gene expression, little is known of how the non-SET domain lysine methyltransferases regulate nuclear receptor function (24). However, recent studies have shown that H3K79 methylation, which is catalyzed by the non-SET methyltransferase DOT1L, is regulated by cell cycle progression (25).

More recently Merm1 was identified in a genetic screen for genes promoting cancer metastases by inhibiting Zac1-mediated p53-dependent apoptosis (26). This action required methylation of histone H3 lysine 9 (H3K9) at the Zac1 locus, thereby rendering a transcriptionally repressive chromatin environment. However, Merm1 did not methylate H3K9 *in vitro*.

Here we show that Merm1 regulates GR binding to its response elements and mediates subsequent H3K4Me3 generation, a mark found on active promoters. Interactome analysis reveals Merm1 binding with histone-associated proteins and protein kinases, intimating a pivotal role for the protein in signal/response coupling in chromatin modeling. Moreover, Merm1 expression was repressed by a combined TNFα/IFNγ proinflammatory environment through an ubiquitination -dependent mechanism, resulting in impaired GR function. Importantly, high level Merm1 expression was found in the bronchial epithelium but was significantly impaired in a broad range of pulmonary inflammatory and neoplastic diseases.

## EXPERIMENTAL PROCEDURES

### 

#### 

##### Plasmids

TAT3-Luciferase (Luc), which contains three copies of the glucocorticoid response element (GRE) from the tyrosine aminotransferase plasmid, was a kind gift from Professor Keith Yamamoto (University of California, San Francisco). The NRE-Luc reporter construct contains five copies of an NF-κB response element (Stratagene, La Jolla, CA). The human wild-type WBSCR22/Merm1 cDNA cloned into cmv.SPORT6 was obtained from Thermo Scientific, Open Biosystems. Three Merm1 deletion constructs were created; ΔMethT, which lacks amino acids (aa) 18–38, ΔSAM, which lacks AA 39–200, and ΔNL, which lacks aa 266–281, were constructed from the wild type by site-directed mutagenesis. The GRIP1 expression vector, in pcDNA3 was kind gift from Dr. Julie Stimmel (GlaxoSmithKline, Research Triangle Park, NC). Renilla luciferase plasmid was used to correct for transfections efficiency (Promega, Southampton, UK). Halo-tag GR and Merm1 were constructed by cloning GR cDNA into the pFN21AB9466 N-terminal vector (Promega). A human mineralocorticoid receptor expression vector was constructed by inserting human mineralocorticoid receptor cDNA into a pcDNAI backbone. Human progesterone receptor B (PR_B_) and androgen receptor cDNAs cloned into PCR3.1 were kind gifts from the Nancy Weigel Laboratory, Baylor College of Medicine (Houston, TX).

##### Antibodies

The following antibodies were used: anti-GR and anti-GRIP1 (BD Biosciences); anti-GR (rabbit, H300 Santa Cruz Biotechnology); anti-GR (rabbit, Novus Biological); anti-Merm1 (WBSCR22) (mouse, Abcam); anti-Merm1 (WBSCR22) (rabbit, Source Bioscience); anti-histone H3 acetyl (rabbit, Millipore); anti-histone H3 lysine 4 trimethyl (rabbit, Millipore) and anti-histone H3 lysine 79 dimethyl (rabbit, Millipore); anti-pan-methyl histone H3 (Lys-9) antibody (rabbit, Cell Signaling); anti-TFIIB (rabbit, Santa Cruz Biotechnology); horseradish peroxidase-conjugated anti-mouse and anti-rabbit from GE Healthcare. Anti-Halo-tag antibody (Promega). Mouse IgG and rabbit IgG were from Millipore. Fluorophore-conjugated (Alexa Fluor 546 and 488) anti-mouse and anti-rabbit antibodies were from Invitrogen.

##### Cell Culture

Human epithelial carcinoma (HeLa), human embryonic kidney cells (HEK293), and human lung carcinoma (A549) cells were obtained from the European Collection of Cell Cultures (Salisbury, UK) and maintained in DMEM supplemented with GlutaMAX I and 10% FBS (Invitrogen) in a humidified atmosphere of 5% CO_2_ at 37 °C.

##### Reporter Gene Assay

Cells were transfected with 1.2 μg of Merm1, GRIP1, or cmv.SP6 plasmid, 2 μg of firefly luciferase (TAT3-Luc), and 0.5 μg of Renilla luciferase reporter using FuGENE 6 (Roche Diagnostics). After 24 h, cells were transferred to medium containing charcoal dextran-stripped serum, treated as specified under “Results,” and then assayed for luciferase activity following the manufacturer's instructions (Promega). To control for transfection efficiency, cells were taken from a single pool and divided into different treatment conditions. All firefly luciferase reading were normalized to Renilla luciferase.

##### Small Interfering RNA (siRNA) Transfection

HeLa cells were transfected with either 10 nm Merm1 siRNA ID s41529 or Merm1 siRNA ID s41530, with appropriate control siRNA 10 nm All Stars Negative Control siRNA (#1027281, Qiagen, Valencia, CA) or Dharmacon siCONTROL Nontargeting siRNA, respectively. Transfection was achieved using Lipofectamine RNAiMax (Invitrogen) or Dharmafect1 (Thermo Scientific) in accordance with the manufacturer's instructions. 48 h later cells were treated as specified under “Results” and processed accordingly.

##### Quantitative RT-PCR

After siRNA and Dex treatment, total RNA was prepared from HeLa cells using the RNeasy mini kit with on-column DNase I digestion (Qiagen), and cDNA was synthesized using a High Capacity RNA to cDNA kit and analyzed using the Power SYBR Green PCR Master Mix (Applied Biosystems). A panel of seven GC target genes was selected from our previous microarray expression studies (27). Quantitative RT-PCR primer sequences are available on request. Expression levels were calculated using the comparative C_t_ method, normalizing to the GAPDH control.

##### Western Blotting

Cells were treated as specified under “Results” and lysed in NETN buffer (0.5% Nonidet P-40, 1 mm EDTA, 50 mm Tris-Cl, pH 8.0, NaCl (120 mm) containing protease (Calbiochem) and phosphatase inhibitors (Sigma). Proteins were separated by SDS gel electrophoresis and transferred to 0.2 μm nitrocellulose membranes (Bio-Rad) overnight at 4 °C. Membranes were blocked for 6 h (0.15 m NaCl, 1% milk, and 0.1% Tween 20) and incubated with primary antibodies (diluted in blocking buffer at 1 in 1000) overnight. After 3 washes (88 mm Tris, pH 7.8, 0.25% dried milk, and 0.1% Tween 20), membranes were incubated with a species-specific horseradish peroxidase-conjugated secondary antibody (in wash buffer) for 1 h at room temperature and washed an additional three times, each for 10 min. Immunoreactive proteins were visualized using enhanced chemiluminescence (ECL Advance, GE Healthcare).

##### Immunofluorescence

Cells were treated as specified and fixed with 4% paraformaldehyde for 10 min at room temperature and permeabilized (0.25% Triton X-100) for 5 min at room temperature. Fixed cells were blocked (3% serum from the species secondary antibody was raised in) for 30 min and then in primary antibody (diluted in blocking buffer) overnight at 4 °C. After three 5-min washes in PBS, cells were incubated in the secondary antibody for 2 h. After three further 5-min washes, coverslips were mounted using Vectashield hard-set mounting compound containing the nuclear DAPI stain (Vector Laboratories). Images were acquired on a Delta Vision RT (Applied Precision, Issaquah, WA) restoration microscope using a ×60/1.42 Plan Apo objective and the Sedat filter set (Chroma 89000; Chroma Technology Corp., Rockingham, VT). The images were collected using a CoolSNAP HQ (Photometrics, Tuscon, AZ) camera with a Z optical spacing of 0.5 μm. Images were deconvolved using Softworx software, and maximum intensity projections of images were processed using Image J.

##### Nuclear and Cytoplasmic Fractionation

Cells were collected and resuspended in ice-cold buffer A (10 mm HEPES, 1.5 mm MgCl_2_, 10 mm KCl, 0.5 mm DTT, 0.05% Nonidet P-40, pH 7.9) supplemented with a mixture of protease and phosphatase inhibitors. Cells were then centrifuged for 10 min at 2000 rpm (291 × *g*). The supernatant was stored as the cytosolic fraction. The pellet was then resuspended in buffer B (5 mm HEPES, 1.5 mm MgCl_2_, 0.2 mm EDTA, 0.5 mm DTT, 26% glycerol (v/v), 300 mm NaCl, pH 7.9) supplemented with protease and phosphatase inhibitors. The samples were incubated on ice for 30 min before centrifugation at 24,000 × *g* for 20 min at 4 °C. The supernatant was kept as the nuclear fraction.

##### Chromatin Immunoprecipitation (ChIP)

For the ChIP assays, cells were grown to a final density of ∼1 × 10^6^ and treated as indicated in the figure legends. ChIP assays were performed as previously described (28, 29). Chromatin was sheared by sonication and size-fractionated to ensure fragmentation to between 200 and 400 bp. For immunoprecipitation, chromatin was incubated with 3 μg of nonspecific IgG or specific antibody at 4 °C overnight. Primers used in the ChIP assay are shown in the supplemental Materials and Methods. Input and immunoprecipitated DNA samples were analyzed and quantified by PCR and gel electrophoresis and by quantitative PCR. Real-time PCRs were carried out in triplicate on each of the immunoprecipitated and input DNA sample. Results are expressed as percentage enrichment relative to input chromatin. Each ChIP experiment was repeated on three occasions, and the mean ± S.D. are shown.

##### Affymetrix Gene Arrays

Human expression data along with accompanying sample descriptions (cell populations are all greater than 95% pure) were purchased from GeneLogic (GeneLogic Division, Ocimum Biosolutions, Inc.) in 2006 and later organized by sample type. Human tissue samples were collected surgically from anonymous donors under Institutional Review Board-approved informed consent. In all cases samples were processed according to rigorous freezing and processing protocols to ensure preservation of RNA and transported to Gene Logic Inc. Clinical medical records were accessioned, reviewed, and entered into Gene Express® software to provide a searchable database of combined clinical, pathological, and gene expression information. Independent pathologists at Gene Logic reviewed each specimen microscopically and required each to be diagnostically accurate for its acceptance and inclusion in the database. Classification as normal was defined as no histopathological abnormality in the tissue under study and no clinical (physical or laboratory finding) abnormality associated with the tissue under study. The tissue type was determined by the surgical pathology report and confirmed microscopically. Expression data for each sample had been determined using mRNA amplification protocols as recommended by Affymetrix (Affymetrix, Inc.) and subsequent hybridization to the Affymetrix U133_plus2 chip. Purchased data were subject to reported quality control measures including minimal 5′/3′ ratios for β-actin and GAPDH as well as maximal scale factors as reported by Affymetrix MAS 5.0. Expression data were normalized using MAS5.0 with a target intensity of 150.

##### Human Embryos

Human embryos were collected with informed consent after ethical approval from the North West Regional Ethics Committee, UK (08/H1010/28) after medical or surgical termination of pregnancy and staged immediately by stereomicroscopy according to the Carnegie classification. The collection, use, and storage of material followed guidelines from the UK Polkinghorne Committee, legislation of the Human Tissue Act 2004, and the Codes of Practice of the Human Tissue Authority, UK.

##### Immunohistochemistry

Paraffin embedded sections were rehydrated (xylene 3 min, 100% for ethanol 2 min, 90% ethanol for 2 min, and a rinse in water), and endogenous peroxidase activity was quenched after a 20-min incubation with H_2_O_2_ (0.1%). After three 5-min washes in PBS antigen was retrieved by boiling in sodium citrate buffer for 20 min, then washed a further 3 times (PBS, 5 min each). Sections were incubated in primary antibody (PBS, 3% goat serum, 0.1% Triton X-100) overnight at 4 °C in a humidified container. After three 5-min washes in PBS, slides were incubated in secondary antibody (1:800 dilution; PBS, 0.1% Triton X-100) for 2 h at 4 °C in a humidified container. After three 5-min washes in PBS, sections were incubated with streptavidin-HRP (1:200; PBS, 0.1% Triton X-100) for 1 h at 4 °C in humidified container and washed a further three times (PBS, 5 min each). Positive immunoreactivity was visualized using 3,3-diaminobenzidine (brown) and nuclei-counterstained with toluidine blue. After a brief wash in PBS, sections were dehydrated (rinse water, 10 s of 70% ethanol, 10 s of 90% ethanol, 3 min of 100% ethanol, 2 min of xylene, 2 min of xylene, 30 s air dry) and mounted using Entellan (Merck Millipore, Intl.). Imaging was performed using an Axioscope Imager A.1 and Axiovision 4.7.1 imaging software (Zeiss).

##### Lung Tissue Array

Human lung disease spectrum tissue array (LC487) was purchased from Insight Biotechnology. The tissue array comprised paraffin-embedded sections (5 μm thick) with duplicate cores per case with two normal lung tissues, one adjacent normal lung tissue, three each of lung hyperplasia of stroma and pulmonary fibrosis with chronic inflammation of bronchiole, one each of lung lobar pneumonia, pulmonary atelectasis, and collapse of lung, two each of pulmonary tuberculosis, pulmonary emphysema, and inflammatory pseudotumor plus one lung small cell carcinoma, two lung adenocarcinomas, and three lung squamous cell carcinomas. Lung sections were processed for immunohistochemistry and stained with anti-Merm1 antibody (rabbit, Source Bioscience; 1:100), and representative images were taken for each core. Four separate images of each section were given a score (1–4) by three independent masked observers. Average scores were calculated and then combined according to pathology.

##### Co-immunoprecipitation of Merm1 and GR Complexes

A549 cells were harvested and homogenized in lysis buffer (20 mm HEPES-KOH, pH 7.4, 0.1 m potassium acetate, 2 mm MgCl_2_, 0.1% Tween 20, 1 μm ZnCl_2_, 1 μm CaCl_2_, 0.5% Triton X-100, 250 mm NaCl, 250 units/sample DNase (Novagen), 1/100 (v/v) protease and phosphatase inhibitor cocktails (Roche)) and centrifuged at 8000 × *g* for 10 min at 4 °C. Lysates were cleared by centrifugation at 13,000 × *g* at 4 °C and incubated with anti-GR antibody (BD Bioscience) for 1 h at 4 °C. The immunoprecipitation was then performed by incubating cell lysates for 30 min at 4 °C with 10 μl/sample of Dynabeads M-280 sheep anti-mouse IgG (Invitrogen). After 3× more washes in lysis buffer, proteins were eluted in NuPAGE LDS sample buffer (4×) (Invitrogen) containing 100 mm DTT (Invitrogen) and incubated for 10 min at 70 °C. Immunoprecipitated proteins were either frozen at −20 °C or used for immunoblot analysis (as described above) using antibodies against GR, phosphorylated GR, and Merm1.

##### Analysis of Merm1 Interacting Proteins

Halo-tag Merm1 constructs were generated from the Merm1-SP6 plasmids according to the manufacturer's instructions (Promega). HEK293T cells were seeded in 20 × 150-mm dishes, 15 μg of Merm1 (N-terminal Halo-tag), or 15 μg of Halo control plasmid, and 45 μg of polyethyleneimine were added to each dish and left for 24 h. The cells (∼10^8^) were washed in PBS and then lysed in Halo lysis buffer (150 mm NaCl, 0.5% Triton X-100, 3 mm MgCl_2_, 20 mm Bicine, 1 μm CaCl_2_, and 1 μm ZnCl_2_, pH 7.4, protease inhibitor, and phosphatase inhibitor. The lysate was passed through a 23-gauge needle and then treated with 100 units of DNase (Promega) for 20 min. The cell lysate was cleared and incubated with Halo-link resin (75 μl/ml, prewashed with (TBS) and 0.05% CA-630 (TBS CA-630) overnight (4 °C). The resin was washed 6 times with TBS CA-630, transferring the resin to a new microtube between each wash. The resin was resuspended in 30 μl of TBS CA-630 with 30 units of tobacco etch virus (TEV) protease and incubated for 2 h on ice, after which 10 μl of 4× SDS (1% SDS and 50 mm Tris-HCL) elution buffer was added. 10 μl of NuPAGE LDS loading buffer and 6 μl of 1 m dithiothreitol was added. Samples were subjected to electrophoresis, and subsequent gels were stained with Simply Blue Coomassie safe stain. Protein bands were excised and destained with repeated incubation in 200 mm ammonium bicarbonate, 40% (v/v) acetonitrile. Gel pieces were dried with 3 washes in 100% acetonitrile and then trypsinized (trypsin resuspended in 100 mm ammonium bicarbonate, 5% (v/v) acetonitrile) overnight at 37 °C. Peptides were extracted from the gel pieces by incubation in 50% (v/v) acetonitrile, 0.1% (v/v) formic acid, and peptides were desiccated and resuspended in 3% (v/v) acetonitrile, 0.1% (v/v) formic acid, 20 mm citric acid, pH 2.7. For each analysis, 10% of the peptide sample was loaded onto a nanoACQUITY UPLC Symmetry C18 Trap (5 μm, 180 μm × 20 mm), and flow was set to 15 μl/min of 3% (v/v) acetonitrile, 0.1% (v/v) formic acid, and 20 mm citric acid for 5 min. Analytical separation of the peptides was performed using a nanoACQUITY UPLC BEH C18 column (1.7 μm, 75 μm × 250 mm). Briefly, peptides were separated over a 91-min solvent gradient from 3% (v/v) acetonitrile, 0.1% (v/v) formic acid to 40% (v/v) acetonitrile, 0.1% (v/v) formic acid on-line to a LTQ Orbitrap Velos (Thermo). Data were acquired using an information-dependent acquisition method where, for each cycle one full MS scan of *m*/*z* 300–1700 was acquired in the Orbitrap at a resolution of 60,000 at *m*/*z* 400 with an automatic gain control target of 106. Each full scan was followed by the selection of the 20 most intense ions; CID (collision-induced dissociation) and MS/MS analysis was performed in the LTQ Orbitrap velos. Selected ions were excluded from further analysis for 60 s. Ions with an unassigned charge or a charge of +1 were rejected.

Data were analyzed using Mascot (Matrix Sciences); the parameters were: Uniprot database, taxonomy *Homo sapiens*, trypsin with up to 1 missed cleavage allowed, variable modifications oxidized methionine, phosphorylated serine, threonine, and tyrosine and the peptide tolerance of 0.025 and 0.03 Da for MS/MS tolerance. A functional annotation of interacting proteins was carried out using the DAVID software package (Version 6.7).

##### Statistical Analysis

Data were expressed as the means ± standard deviation and compared using the SPSS software package (Version 16, SPSS Inc., Chicago, IL). Multiple means were compared by one-way ANOVA followed by the Bonferroni post hoc test, Tukey's test, or Kruskal-Wallis test, and for comparison of two groups, Student's *t* test or the Mann Whitney *U* test (with a Bonferroni correction where appropriate) for independent samples was used. *p* ≤ 0.05 was considered statistically significant.

## RESULTS

### 

#### 

##### Merm1 Regulates GR Transactivation

**FIGURE 1.**
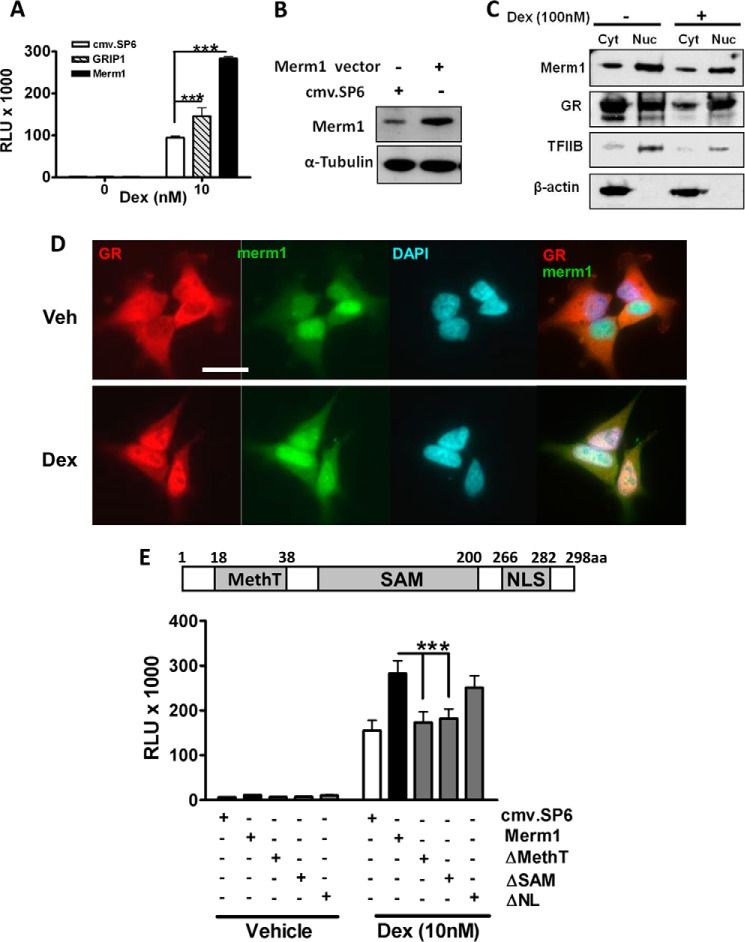
**Merm1 enhances GR-mediated transactivation via its SAM and methyltransferase domains.** *A*, HeLa cells were transfected with 2 μg of firefly luciferase (TAT3-Luc) and 0.5 μg of Renilla luciferase reporter with one of Merm1, GRIP1, or cmv.SP6 plasmid (1.2 μg) as indicated and incubated with 10 nm Dex for 24 h before luciferase assay. Graphs depict the means ± S.D. of triplicate wells from three independent experiments. Data were compared using one way ANOVA followed by Bonferroni's post hoc test. *B*, cell lysates prepared from *A* were used to confirm induction of Merm1 expression. Cell lysates were resolved by SDS-PAGE, and after transfer to nitrocellulose membranes were analyzed with a specific Merm1 antibody, and tubulin was used as a loading and transfer control. *C*, HeLa cells were treated with either vehicle or 100 nm Dex for 1 h. They were then lysed and separated into cytosolic (*Cyt*) and nuclear (*Nuc*) fractions. Cellular fractions were separated by gel electrophoresis and immunoblotted using antibodies against β-actin (cytosol), TFIIB (nuclear), GR, and Merm1. Experiments were repeated three times with similar results. *D*, HeLa cells were treated with vehicle (*Veh*) or 100 nm Dex for 1 h, then fixed and labeled with antibodies specific to Merm1 (*green*) and GR (*red*). Representative images are shown. *Scale bar*, 50 μm. *E*, HeLa cells were transiently co-transfected with the TAT3-Luc reporter gene, Renilla control plasmid, and the indicated Merm1 expression plasmids (wild type, ΔMethT, ΔSAM, ΔNL). Diagrammatic representation of Merm1 protein and its functional domains (with amino acid numbers) is shown. Within the indicated SAM domain is a highly conserved D*X*G*X*G*X*G*XX*G-like motif where *X* may be any amino acid, and D and G are aspartic acid and glycine, respectively. *NLS*, nuclear localization signal. The graphs show mean ± S.D. of triplicate wells. Experiments were repeated on three occasions. *RLU*, relative light unit; ***, *p* < 0.001. Analysis by ANOVA was followed by the Bonferroni test.

We have previously used a stratified screening approach to identify genes capable of modulating GR function (27, 30). Here, we profiled Merm1 because of its putative action as a histone methyltransferase and its action as a transcriptional co-repressor (26). Unexpectedly, we found that overexpression of Merm1 (Fig. 1, *A* and *B*) potentiated GR transactivation of a TAT3-Luc reporter gene in a similar manner to GRIP1 (Fig. 1*A*). Merm1 protein was found to be endogenously expressed in HeLa cells (Fig. 1*B*), permitting analysis of cellular distribution and trafficking (Fig. 1, *C* and *D*). Merm1 localization was initially studied using biochemical means in fractionated cells with β-actin as a cytoplasmic and TFIIB as a nuclear marker. Merm1 was distributed in both nuclear and cytoplasmic compartments and showed no alteration in distribution with GC treatment, although as expected the GR accumulated in the nucleus of treated cells (Fig. 1*C*).

Further analysis of Merm1 distribution using immunofluorescent approaches supported the subcellular fractionation results (Fig. 1*D*). Again Merm1 was seen through both cytoplasmic and nuclear compartments, with no change seen after GC treatment. GR translocation was observed as expected, serving as a useful internal control (Fig. 1*D*). The overlay pictures show stronger overlap of Merm1 and GR distributions after GC treatment, as evidenced by the increase in yellow immunofluorescence (Fig. 1*D*, *right panels*).

GR transrepression was investigated using a TNFα-activated NF-κB reporter gene in HeLa cells. In this model TNFα drives activation of an NF-κB reporter gene, and this induction is repressed by activated GR. Neither Merm1 nor GRIP1 affected GR transrepression under these conditions (supplemental Fig. S1). Merm1 potentiated transactivation by the related mineralocorticoid receptor, progesterone receptor, and androgen receptor on the same TAT3-Luc reporter gene when transfected with the appropriate nuclear receptor expression vectors in the nuclear receptor-deficient cell line HEK293T (supplemental Fig. S2). This observation demonstrates broad Merm1-mediated steroid receptor co-activation.

##### Functional Domain Analysis of Merm1 for Regions Responsible for Potentiating GR Transactivation

Merm1 contains a highly conserved methyltransferase domain (MethT), an *S*-adenosyl-l-methionine (SAM) domain, and a nuclear localization signal (NLA) domain (31) (Fig. 1*E* and supplemental Fig. S3). To determine which of the functional domains is responsible for the potentiation of GR transactivation, deletion constructs were generated in which individual functional domains (as indicated in Fig. 1*E*) were deleted. The Merm1 ΔNL construct still potentiated the glucocorticoid-induced transactivation in HeLa (*p* < 0.001) cells, whereas deletion of either the SAM or MethT domains abolished the co-activation function of Merm1. Expression of the mutated constructs was analyzed by immunoblotting (supplemental Fig. S3). The SAM domain mutant is heavily truncated and may not be detected by the Merm1 antibody (raised against amino acids 1–263 (Fig. 1*E* and supplemental Fig. S3).

##### Merm1 Is Widely Expressed, Showing High Levels of Expression in Bronchial Epithelium and Transcript Induction in Activated CD8+ T and B Lymphocytes

**FIGURE 2.**
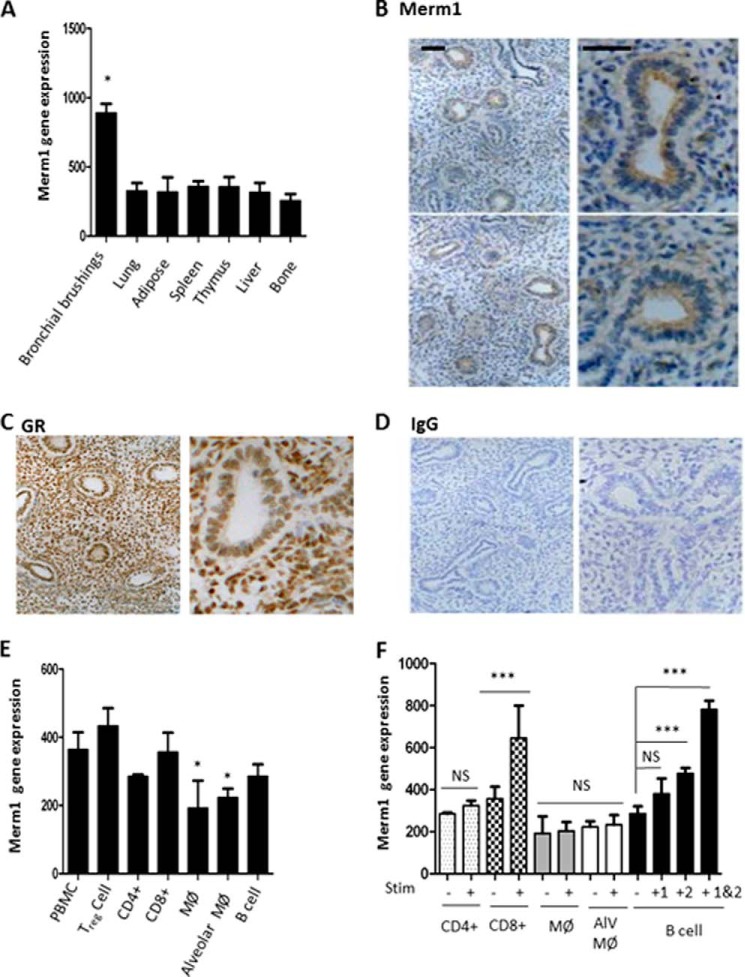
**Merm1 expression in normal human tissues.** *A*, the expression of Merm1 was measured in a profile of normal human tissue samples acquired and analyzed by Affymetrix gene array. Sample preparation and analysis is described under “Experimental Procedures.” Independent samples were used: bronchial brushings = 7; lung = 100; spleen = 31; adipose = 113; thymus = 67; liver = 32; bone = 12. Analysis was carried out using a one-way ANOVA, with Tukey's post-hoc test (*, *p* < 0.05). *B–D*, Merm1 and GR expression in human fetal lung. Normal, human fetal lung explants were prepared, fixed, and analyzed by immunohistochemistry. Antibody binding was disclosed by 3,3-diaminobenzidine staining (*brown*), with nuclei counter-stained with toluidine blue. *E*, Merm1 gene expression in hematopoietic cells. Human Affymetrix gene expression databases (see “Experimental Procedures”) were interrogated for Merm1 expression. Independent samples were analyzed; *n* = 4, except alveolar macrophages, *n* = 5, and macrophages, *n* = 9. *, *p* < 0.05 one way ANOVA followed by Bonferroni test. *F*, Merm1 expression in stimulated hematopoietic cells. Human Affymetrix gene expression databases (“Experimental Procedures”) were interrogated for Merm1 expression. CD4 and CD8 lymphocytes were stimulated with anti CD3 (*n* = 4 for each group), macrophages (*M*Ø) were stimulated with LPS (6 h treatment) (*n* = 9 for each group); alveolar macrophages (*Alv M*Ø) were unstimulated (*n* = 5) and stimulated with LPS (6 h *n* = 15); B cells were stimulated with CD40 ligand (*1*), anti-B cell receptor antibody (*2*), or both (48 h treatment) (*n* = 4 for each group). ***, *p* < 0.001, one-way ANOVA, with Bonferroni post-hoc test; *NS*, not significant.

To guide further the investigation of Merm1 function, we determined Merm1 tissue distribution using Affymetrix array profiles of normal human tissue. These databases were derived from human tissue samples, collected under standardized conditions, and analyzed by Affymetrix gene arrays. Interrogation of these databases revealed near ubiquitous Merm1 expression, but a striking finding was greatly increased expression in human bronchial brushings, a mixture of cell types that line the major airways including ciliated epithelial cells, Clara cells, goblet cells, and airway resident macrophages (Fig. 2*A*). To determine if this pattern of gene expression resulted in a distinct pattern of Merm1 protein expression, we analyzed human fetal lung tissue. Expression of Merm1 protein (brown) was seen to be most obvious in the developing bronchial lumen lining cells (Fig. 2*B*), a result compatible with the gene expression data above (Fig. 2*A*). By comparison, expression of GR protein was seen more diffusely through the lung architecture (Fig. 2*C*). Use of IgG controls revealed negligible background staining (Fig. 2*D*). As inhaled GC is a widely used intervention for inflammatory lung disease but suffers from a wide variation in response, we measured Merm1 expression and regulation in a variety of relevant immune cell types under basal and activated conditions (Fig. 2, *E* and *F*). Again, we interrogated commercially available Affymetrix gene expression databases (see “Experimental Procedures”). Primary human immune cells were purified from blood or lung (as indicated) and verified to be at least 95% pure based on analysis of cell surface markers. Cells were activated by the indicated stimuli and time before analysis.

The most striking feature seen was augmented Merm1 transcript abundance in CD8+ T lymphocytes and B lymphocytes when activated with the classical stimuli CD3 for CD8+ T and CD40 ligand and B cell receptor antibody for B lymphocytes (Fig. 2, *E* and *F*). Merm1 expression in macrophages, either monocyte-derived macrophages (MØ) or alveolar macrophages (ALV MØ), was not affected by activation with LPS (Fig. 2*F*).

##### Analysis of the Merm1 Protein Interactome

**FIGURE 3.**
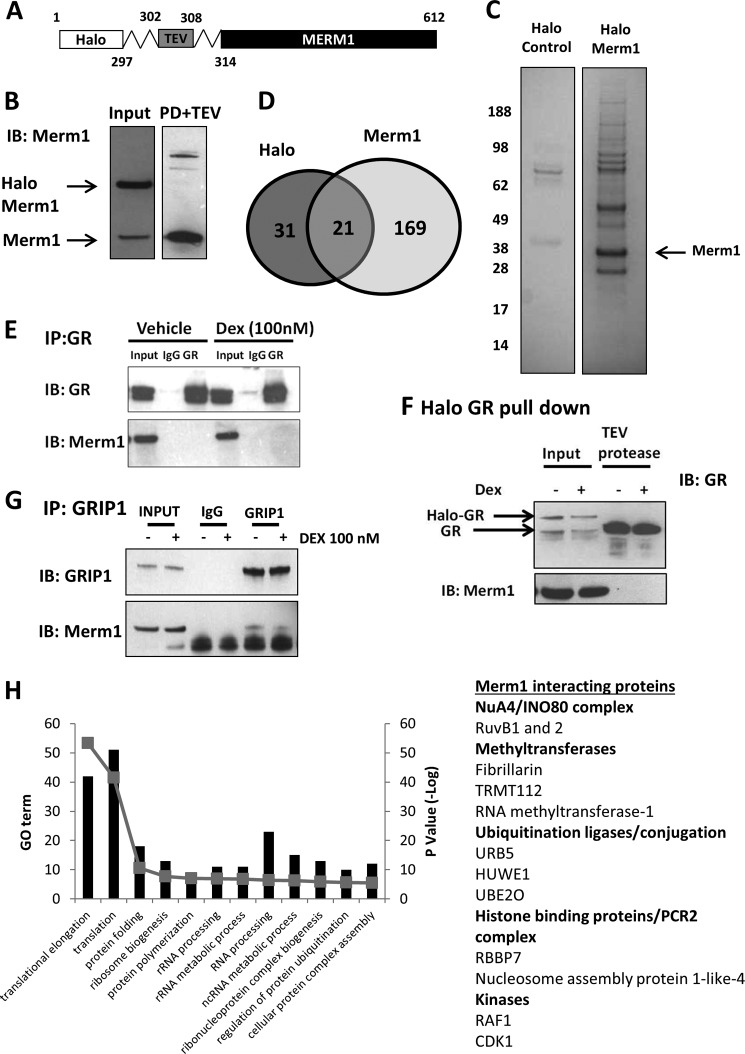
**Analysis of the Merm1 interactome.** *A*, the Halo-tag is a 34-kDa protein tag from dehalogenase (*Rhodococcus* sp.) which binds to its ligand covalently. Halo-Merm1 is captured with the Halo-link resin. Merm1 is cleaved from the Halo ligand (leaving the Halo-tag on the resin) complex using a TEV protease, which targets a protein sequence at the linker as indicated. Eluted proteins were then analyzed by SDS-PAGE and mass spectrometry. Immunoblots showing Halo-control and Halo-Merm1 expression in HEK cells; PD+TEV (pull down and TEV) refers to the sample after it had been bound to the Halo-link resin, washed, and treated with the TEV protease. Approximately 1% of the sample was loaded for immunoblot. The same process was performed for the Halo-control protein. *B*, Halo-Merm1-transfected cell lysate (*Input*) and eluate after Halo-resin purification of the transfected cell lysate and subsequent TEV cleavage (PD+TEV) were resolved by SDS-PAGE and immunoblotted (*IB*) for Merm1. The migration of Halo-tagged Merm1 and full-length Merm1 are indicated (based on molecular weight and antibody reactivity). *C*, HEK293T cells were transfected in bulk to permit proteomic analysis of Merm1 interacting proteins. For both Halo control and Halo-Merm1, 10 × 15-cm dishes in total were transfected before being pooled for analysis. Proteins eluted from the Halo resin post cleavage with TEV were resolved by SDS-PAGE, and the gels were stained with Coomassie Blue. Molecular mass marker migration is indicated. *D*, proteins identified by mass spectrometry that were unique for the Halo control (*n* = 31), Halo-Merm1 (*n* = 169), and proteins enriched under both conditions (*n* = 21) (using the ensembl gene ID) are presented in Venn diagram. *E*, A549 cells were treated with either vehicle or 100 nm Dex for 1 h. Cells were lysed and immunoprecipitated (*IP*) with antibodies against GR. Mouse IgG was used as the control for the IP assay. Precipitates were immunoblotted for GR, and Merm1. *F*, A549 cells were transfected with 0.4 μg/ml Halo-tag-GR or Halo-tag control vector. A549 cells were treated with vehicle or 100 nm Dex for 1 h and then lysed and bound to Halo link resin. The Halo-tag was removed as described above. Precipitates were immunoblotted for GR and Merm1. *Input lanes* reveal slower migration of the Halo-tagged GR. *G*, as *E*, but immunoprecipitation using a GRIP1 antibody. Immunoprecipitates were analyzed with antibodies to GRIP1, and Merm1. *H*, Merm1 interacting proteins were analyzed with the DAVID bioinformatics software (Version 6.7); the top 12 gene ontologies (*GO term*) are shown as is the *p* value (shown as −*Log*). A list of notable Merm1 interacting proteins was manually curated (see supplemental Table 1 for full details).

To assist in determining the mechanism of Merm1 co-activation of the GR, we mapped the Merm1 interactome, analyzing purified Merm1 complexes by mass spectroscopy (Fig. 3). To generate a robust and clean Merm1 bait, we used a Halo-tagged Merm1 expression cassette. The Halo-tag (Fig. 3*A*) is recognized by an immobilized ligand, thereby capturing Merm1 complexes for proteomic analysis. This offers advantages over antibody purification protocols by minimizing off-target protein capture. All analyses were controlled using the Halo-tag alone to permit subtraction of proteins recognizing the epitope tag.

The Halo-tagged Merm1 was transfected into HEK293T cells, which offer high transfection efficiency and high transfected protein expression. The cellular expression of Halo-tagged Merm1 was verified using an immunoblot of transfected cells (Fig. 3*B*, *input lane*) and the successful cleavage of the Halo-tag by use of the TEV from the pulldown material (*PD*), and thereby enrichment of Merm1 protein was also confirmed by immunoblot (Fig. 3*B*, *PD*+*TEV lane*).

Purified Halo complexes (Halo-tag alone or Halo-Merm1) were resolved by SDS-PAGE (Fig. 3*C*) and subject to mass spectroscopy protein identification. Proteins identified by that were unique for the Halo-Control (*n* = 31), Halo-Merm1 (*n* = 169), and proteins enriched under both conditions (*n* = 21) (Fig. 3*D*).

Previous studies had failed to detect intrinsic Merm1 histone methyltransferase activity in reconstituted *in vitro* assays. We were also unable to detect direct histone methyltransferase activity and postulated a requirement for additional, unknown cellular components for Merm1 catalytic action (26). We did not detect an interaction between GR and Merm1, which may reflect the sensitivity limits of our proteomics technology but may also result from low level GR expression in HEK293T. Therefore, we sought evidence of interaction using immunoprecipitation-immunoblotting in A549 cells (Fig. 3*E*). As demonstrated above, A549 cells express endogenous GR and Merm1 and, moreover, possess the machinery to permit Merm1 co-activation of the GR. Immunoprecipitation of GR from vehicle and GC-treated A549 cells was successful, and although Merm1 was seen in the input cell lysate no detectable band was seen in the GR immunoprecipitate (Fig. 3*E*). This provides additional evidence that Merm1 does not physically bind to GR, but in a further attempt to detect interaction we used a Halo-GR fusion protein as bait. A549 cells were transfected with a Halo-GR expression vector, and Halo complexes were purified as above. Both endogenous GR and Halo-GR were detectable in the input samples (Fig. 3*F*, *input lanes*). After TEV cleavage, abundant full-length GR was seen in the eluate (Fig. 3*F*, *TEV protease lanes*).

The lack of evidence to support an interaction between GR and Merm1 suggested an indirect mechanism of regulation. A strong candidate is the GR co-activator and scaffold protein GRIP1/SRC2. Therefore, we performed immunoprecipitation-immunoblot studies in A549 cells with GRIP1. We were able to enrich GRIP1 in cell lysates under vehicle and GC-treated conditions (Fig. 3*G*) and were also able to detect a weak interaction with Merm1 as detected by blotting the immunoprecipitate (Fig. 3*G*).

Among the interacting partners we identified (Fig. 3*H*, supplemental Table 1 and supplemental data) were proteins with a functional role for Merm1 in driving histone modification and ribosome biogenesis (32), including the histone-binding protein RBBP7, which binds H3K4 in a methylation-dependent manner (33), and RUVBL1 and -2, which are both components of the NuA4 and INO80 complexes (Fig. 3*H* and supplemental Table 1). Merm1 was also found with ubiquitin protein ligases, and in addition, ubiquitin was found in the protein complex, suggesting a potential mechanism for regulating Merm1 protein levels by ubiquitinylation and degradation by the 26 S proteasome. Many of the identified Merm1 interacting proteins contribute to ribosome biogenesis, as shown in the top 12 gene ontologies (Fig. 3*H*). We also found TRMT112, the human homologue of TRM112, which has been shown to be essential for the stability of the yeast WBSCR22 homologue (Bud23) (34).

##### Merm1 Alters GR Target Gene Expression by GR-dependent and -independent Mechanisms

**FIGURE 4.**
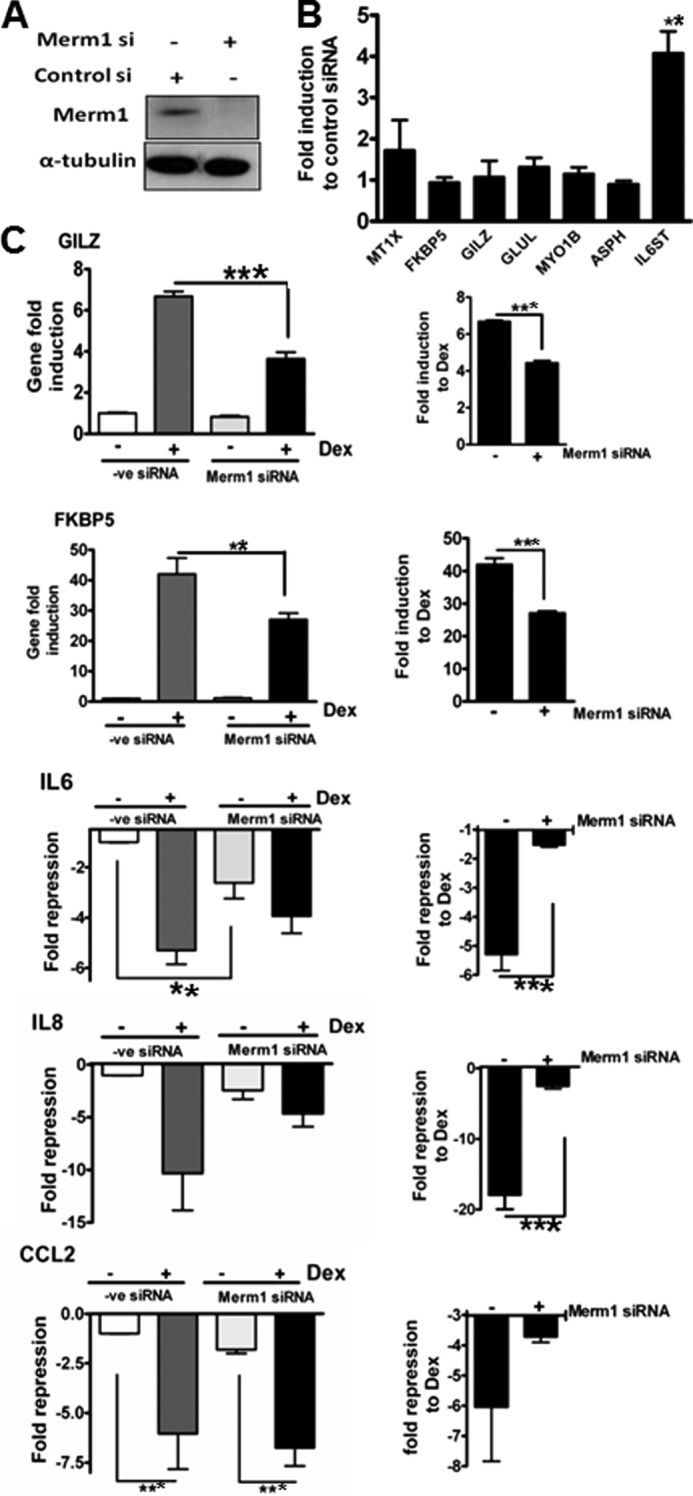
**Merm1 affects GR regulation of endogenous genes.** *A*, HeLa cells were transfected with either 10 nm Merm1 or control siRNA for 48 h before immunoblotting for Merm1 and α-tubulin. Representative images are shown. *B*, HeLa cells were transfected with 10 nm Merm1 or control siRNA as in *A*. Post lysis RNA was purified, and quantitative RT-PCR was used to measure basal expression of seven endogenous index glucocorticoid-regulated genes (aspartate β hydroxylase (*ASPH*), FK506-binding protein 5 (*FKBP5*), metallothionine 1X (*MT1X*), interleukin-6 signal transducer (*IL6ST*), myosin 1β (*MYO1B*), glutamate ammonia ligase (*GLUL*), and glucocorticoid leucine zipper (*GILZ*)). Experiments were performed in triplicate on three occasions. Data presented are the mean ± S.D., and analysis in one way ANOVA was followed by Bonferroni tests. **, indicates *p* < 0.01 compared with control siRNA. *C*, HeLa cells were transfected with 10 nm Merm1 or control siRNA for 48 h and then treated with vehicle or 100 nm Dex for 6 h before lysis and RNA extraction. The expression of index, endogenous GR target genes GILZ, FKBP5, IL6, IL8, and CCL2 was measured by quantitative RT-PCR. Gene expression is expressed as -fold change. For induced genes the post Dex expression is divided by the negative control (−ve). For repressed genes the control expression is divided by the post Dex expression (shown as a negative number). On the *left* all the data are expressed relative to the control siRNA, vehicle-treated group, and on the *right* the same data are plotted to show the effect of Dex as a -fold change under control siRNA or specific Merm1 siRNA-treated conditions. Graphs show the mean ± S.D. of experiments performed in triplicate and are repeated three times. **, *p* < 0.01; ***, *p* < 0.001, analysis was by one way ANOVA and post hoc Bonferroni tests.

To identify endogenous gene targets for Merm1, siRNA knockdown was used in A549 cells. Merm1 expression was virtually abolished by siRNA (Fig. 4*A*). Seven glucocorticoid-regulated genes (27) were selected for initial analysis. Merm1 knockdown augmented base-line expression of one of the seven genes, interleukin-6 signal transducer (*IL6ST*) (Fig. 4*B*). TSC22 domain family 3 (*GILZ*, *TSC22D3*) and FK506-binding protein 5 (*FKBP5*) were not regulated by Merm1 loss alone, but in the absence of Merm1 both FKBP5 and GILZ were significantly less responsive to GR induction (Fig. 4*C*). For both genes, again, loss of Merm1 had no effect on basal expression (Fig. 4*C*).

In addition to these effects on GR transactivated target genes, we analyzed three proinflammatory cytokine/chemokine genes: interleukin 6 (IL6), interleukin 8 (IL8), and chemokine (C-C motif) ligand 2 (*CCL2*) (Fig. 4*C*). Even in the absence of cell activation GC treatment significantly inhibited expression of all three genes (Fig. 4*C*).

**FIGURE 5.**
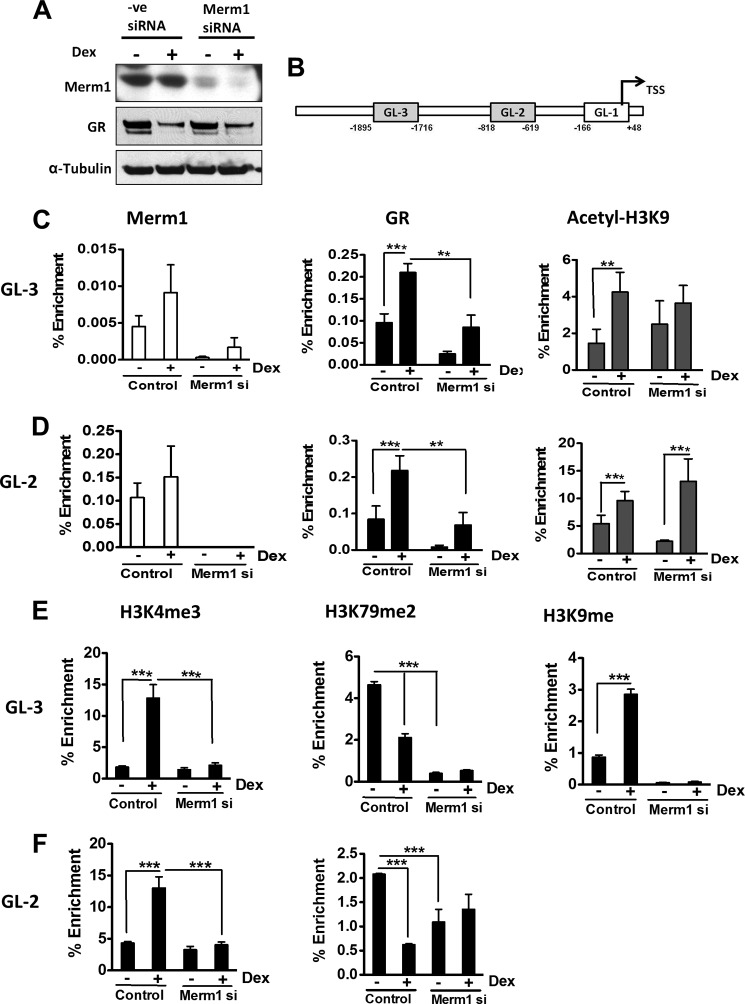
**Merm1 and GR ChIP to the same region of GILZ promoter and regulates histone methylation.** *A*, HeLa cells were grown and transfected with 10 nm Merm1 specific siRNA (*Merm1 siRNA*) or control siRNA (control) for 48 h. Cells were treated with either vehicle or 100 nm Dex overnight and then lysed and immunoblotted for Merm1, GR, and α-tubulin. *B*, schematic representation of GILZ promoter upstream of transcription start site (*TSS*). The *gray boxes* represent the regions with GREs. GL-1, -2, and -3 are primer pairs used in the ChIP assay. GL-1 served as the non-GR binding (negative) control region for the ChIP assay. GL2 and GL-3 contained 1 and 3 characterized GREs, respectively (31). The transcriptional start site is shown by the *black arrow. C* and *D*, control and siRNA-mediated Merm1 knocked-down HeLa cells were treated with vehicle or 100 nm Dex for 1 h. They were then fixed using formaldehyde, and a ChIP assay was performed using antibodies against GR, Merm1, and acetylated H3K9 and analyzed with primers for GL-3 (*C*) and GL-2 (*D*) promoter regions. *E* and *F*, ChIP assays were performed using antibodies against H3K4me3, H3K79me2, and H3K9me with primers for GL-3 (*E*) and GL-2 (*F*). Control and Merm1-specific siRNA were used as indicated. Nonspecific rabbit and mouse IgGs were used as controls for the immunoprecipitation assay. The recovered chromatin fractions were analyzed both by PCR and agarose gel electrophoresis and also by quantitative PCR for precise quantification. Results are expressed as a % enrichment of recovered immunoprecipitated chromatin relative to the input sample. All ChIP assays were performed on three separate occasions using cells of different passage before the data were pooled for analysis. Graphs show the mean ± S.D. **, *p* < 0.01 and ***, *p* < 0.001.

Loss of Merm1 expression alone reduced expression of IL-6, suggesting coordinate regulation of the IL-6 autocrine circuit, as the IL6 signal transducer is induced by Merm1 loss (Fig. 4, *B* and *C*). In contrast, IL8 and CCL2 were unaffected by loss of Merm1 under basal conditions (Fig. 4*C*, *left panels*). However, loss of Merm1 impaired glucocorticoid-repression of both IL6, and IL8, but had no effect on CCL2 (Fig. 4*C*, *left panels*). Importantly, Merm1siRNA did not affect GR expression (Fig. 5*A*). As a further control for the specificity of the Merm1 siRNA, we used a different Merm1 siRNA, which caused a similar loss of GR transactivation (supplemental Fig. S4–S6). This selectivity of target gene regulation suggests a strong role for target gene specific factors in conferring Merm1 co-modulation to the GR.

##### Merm1 and GR Locate to the Same Region of GILZ Promoter

To determine how Merm1 regulates GR transcriptional activity, we employed the well characterized “index” glucocorticoid-regulated gene, GILZ. Crucially, GILZ gene expression is not affected by Merm1 knockdown alone, and the binding sites conferring GR transactivation have been previously mapped (35). We used the endogenous GILZ gene as a template to analyze GR gene regulation, employing a ChIP approach targeting the two characterized GR binding sites and using the non-GR binding region around the transcription start site (*GL-1*) as control (Fig. 5*B* and supplemental Fig. S7).

Under basal conditions neither GR nor Merm1 was bound to GL-1. Merm1 was bound to GL-2 under basal and GC conditions but was barely detectable on GL-3. GR loading on GL-2 and GL-3 regulatory regions was significantly increased by GC (Fig. 5, *C* and *D*), with attendant induction of H3K9 acetylation at both sites examined (Fig. 5, *C* and *D*). Knockdown of Merm1 with siRNA resulted in a significant loss of GR loading onto both the GILZ GREs (GL-2 and GL-3) as measured by quantitative PCR of immunoprecipitated chromatin compared with input (Fig. 5, *C* and *D*). There was detectable GR loading to both GL-2 and GL-3 under basal conditions, and loss of Merm1 expression reduced this loading to a greater extent for GL-2 than GL-3. The functional significance of this low level GR loading is unclear, but the small reduction in histone H3K9 acetylation seen at GL-2 supports a productive interaction with GR at this locus even under ligand-free conditions, which is dependent on Merm1.

##### Merm1 Modulates Histone Methylation in Response to GR Activation

As Merm1 has putative histone methyltransferase activity, we analyzed histone H3 methylation in response to activated GR (Fig. 5, *E* and *F*). After GC treatment, there was a marked induction of the active promoter chromatin mark H3K4me3 at GL-2 and GL-3 (Fig. 5, *E* and *F*) and reduction of the elongation chromatin mark H3K79me2 (Fig. 5, *E* and *F*). In addition, we documented a small, but significant induction of the repressive mark H3K9me at GL-3 in response to GC (Fig. 5*E*), with no detectable H3K9me at GL-2 under any condition examined. Overall the H3K4Me3 mark was dominant, with near 15% enrichment compared with <5% for H3K79Me2 and H3K9me.

The GR-mediated induction of H3K4me3 was dependent on Merm1 expression (Fig. 5, *E* and *F*). However, Merm1 was also found to mediate basal H3K79me2 (Fig. 5, *E* and *F*). After activation of GR, this methylation mark was reduced, again in a Merm1-dependent manner. The H3K9me mark was abrogated at GL-3 in the absence of Merm1 (Fig. 5*E*). These results support the existence of a coordinated histone methylation response to GR recruitment that is mediated at least in part by Merm1.

##### Merm1 Mediates Cytokine-induced Resistance to Glucocorticoid

**FIGURE 6.**
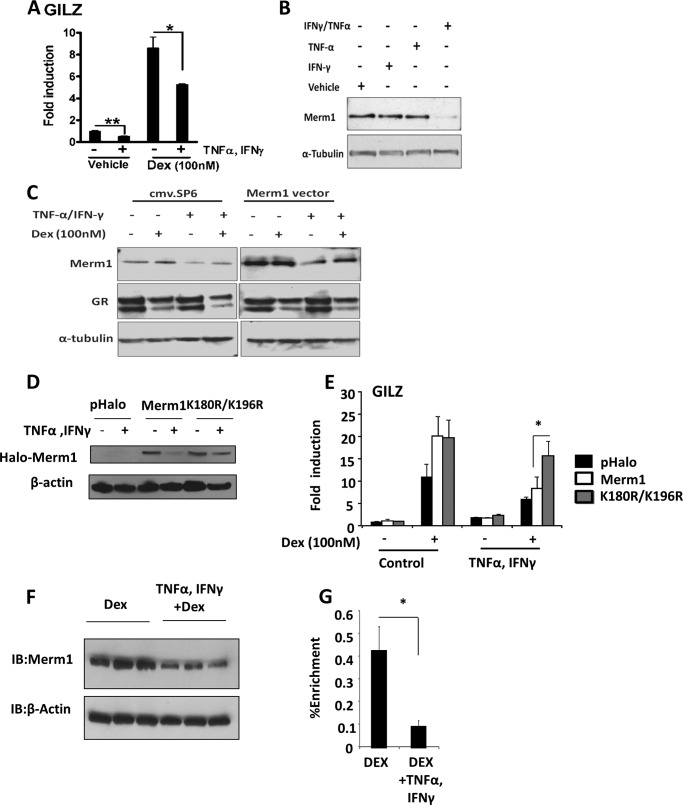
**Merm1 mediates cytokine-induced glucocorticoid resistance.** *A*, A549 were treated with 10 ng/ml TNFα and 500 units/ml IFNγ for 16 h. 100 nm Dex was added for a further 6 h as indicated before RNA extraction. GILZ transcript abundance was measured by RT-PCR and is expressed as -fold induction from control cells. Graphs show the mean ± S.D. (*n* = 3). *, *p* < 0.05, one way ANOVA followed by Bonferroni test. *B*, A549 cells were treated with either 10 ng/ml TNFα or 500 units/ml IFNγ alone or both TNFα and IFNγ in combination for 16 h. Cells were lysed and immunoblotted for Merm1 and tubulin expression. The experiment was performed on three occasions with similar results. *C*, A549 cells were transfected with 1.2 μg of Merm1 expression vector or empty plasmid (*cmv.SP6*). After overnight incubation, cells were treated with 10 ng/ml TNFα and 500 units/ml IFNγ for another 16 h. Cells were treated with 100 nm Dex for 6 h before harvest. Under these conditions lysates were analyzed by immunoblot for Merm1, GR, and tubulin expression. *D*, A549 cells were transfected with 1.2 μg of WT Halo-Merm1 expression vector, K180R/K196R Merm1, or empty plasmid (*pHalo*). After overnight incubation, cells were treated with 10 ng/ml TNFα and 500 units/ml IFNγ for another 16 h and then harvested for immunoblot analysis of Halo-Merm1 protein. Experiments were repeated on three occasions with similar results. *E*, A549 cells were transfected with 1.2 μg of WT Halo-Merm1 expression vector, K180R/K196R Merm1, or empty plasmid (*pHalo*) as in *D*. After overnight incubation, cells were treated with 10 ng/ml TNFα and 500 units/ml IFNγ for another 16 h. Cells were treated with Dex 100 nm as indicated. After lysis GILZ mRNA was quantitated by quantitative RT-PCR. Graphs show the mean ± S.D. (*n* = 3). *, *p* < 0.05 using an independent Student's *t* test. *F*, human fetal lung explants were treated with TNFα and IFNγ (as described above) and immunoblotted (*IB*) for Merm1 and β-actin. Triplicate experiments are shown. *G*, human fetal lung was treated as in *F* and GR ChIP was performed using primers for the GL-3 region of GLIZ promoter (as in Fig. 5). Data are presented as the mean ± S.D. (*n* = 6). Statistical significance was determined using a Mann Whitney test. *, *p* < 0.05.

Merm1 expression may be a mechanism to regulate GR function both by controlling GR access to target sites and by affecting or executing GR-directed methylation of histones. To investigate possible physiological signals regulating Merm1 expression, a panel of stimuli was screened, including cytokines and growth factors. Of these the proinflammatory cytokines TNFα and IFNγ were tested in combination, as they are together known to render target cells insensitive to GC (36) (Fig. 6*A*). Incubation with either cytokine alone was insufficient to affect Merm1 expression, but both together significantly reduced Merm1 protein levels (Fig. 6*B*).

In an attempt to restore glucocorticoid sensitivity, Merm1 was overexpressed in the presence of TNFα and IFNγ. Transfection with a Merm1 expression vector greatly increased Merm1 protein concentration, but treatment with combined TNFα/IFNγ still reduced Merm1 protein accumulation (Fig. 6*C*).

This reduction in Merm1 protein from both endogenous and ectopic expression suggested a post-translational mechanism of regulation (Fig. 6*C*). We had earlier identified ubiquitin and ubiquitinylation enzymes within the Merm1 interactome, and reports had suggested potential sites for ubiquitinylation on Merm1 (37, 38). Mutation of these two lysines (K180R/K196R) rendered Merm1 resistant to degradation in response to the combined proinflammatory cytokines (Fig. 6*D*), and moreover, the mutated Merm1 protein was a more effective co-activator for GR than wild-type Merm1 in the presence of TNFα/IFNγ, potentially by enhanced expression or stability within the complex (Fig. 6*E*).

The discovery of a pathway linking proinflammatory cytokines through loss of Merm1 protein to impaired GR function in cells prompted us to investigate human lung tissue, a prominent site of Merm1 expression. Indeed, in human fetal lung explants combined IFNγ/TNFα also suppressed Merm1 protein levels (Fig. 6*F*). As predicted, this was accompanied by loss of GR recruitment to the consensus GRE upstream of the GILZ gene transcription start site (Fig. 6*G*).

##### Merm1 Expression Is Suppressed in Human Lung Diseases Associated with GC Resistance

**FIGURE 7.**
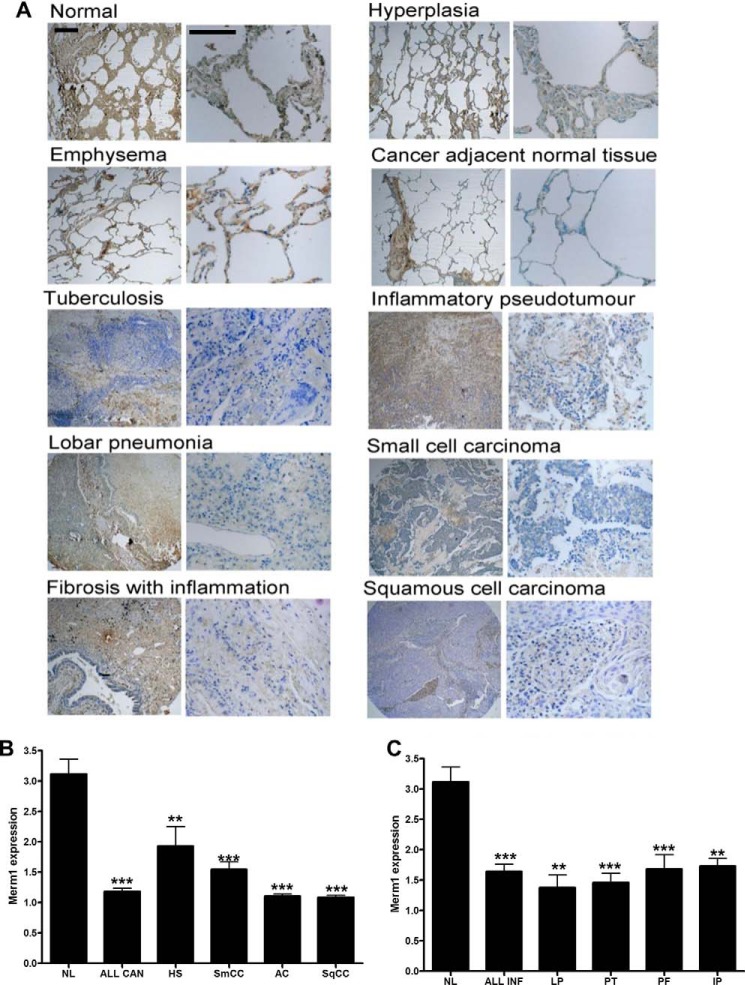
**Merm1 expression in Lung diseases.** *A*, Merm1 expression in a human lung disease tissue array. Human lung sections from Insight Biotechnology (LC487) were analyzed for Merm1 protein expression by immunohistochemistry; anti-Merm1 antibody (1:100). Low and high magnification views are presented for the different pathological states analyzed. The expression of Merm1 in each core sample was estimated by three masked observers scoring four microscope fields for each core. *Scale bar*, 100 um). Specific antibody binding was disclosed by 3,3-diaminobenzidine (*brown*) and nuclei counterstained with toluidine blue. *B* and *C*, the quantification of Merm1 expression seen in the different pathological states in (*A*) is presented. Expression was estimated on an arbitrary scale from 1–4, with 4 being very intense staining. Cancer pathology is presented in *B*, and inflammatory pathology is presented in *C. NL*, normal lung; *All Can*, all cancer states combined; *HS*, hyperplasia of the stroma; *SqCC*, squamous cell carcinoma; *AC*, adenocarcinoma; *SmCC*, Small cell carcinoma. *C*, normal lung repeated from *B. All infect*, all infection scores were combined; *LP*, lobar pneumonia; *PT*, pulmonary tuberculosis; PF, pulmonary fibrosis; *IP*, inflammatory pseudotumor. Analysis was by one-way ANOVA with Bonferroni post hoc tests. **, *p* < 0.01, ***, *p* < 0.001 compared with normal lung.

To establish if these findings are clinically relevant we addressed expression of Merm1 protein in human lung pathologies to test the hypothesis that inflammatory processes would be associated with loss of Merm1 protein. A human pathological lung tissue microarray was analyzed for Merm1 immunoreactive intensity by three masked observers (Fig. 7). Representative sections are shown (Fig. 7*A*) with quantification given by disease type (Fig. 7, *B* and *C*). Inflammatory (Fig. 7*B*) and neoplastic lung pathologies (Fig. 7*C*) were grouped revealing a significant loss of Merm1 protein expression associated with both disease processes.

## DISCUSSION

Recent discoveries have identified the importance of chromatin architecture for directing GR to its binding sites, therefore offering an explanation for tissue-specific actions of a ubiquitously expressed transcription factor (10, 39). In addition, it has been recognized that the actions of the GR to regulate target gene transcription are, in part, mediated by directed chromatin remodeling, with resulting changes in the access to, and binding of other transcription factors (15, 40–43). Most recently it has become clear that GR and other transcription factors may associate with their binding sites in a highly dynamic state (44–46). Therefore, much interest has been directed at understanding how GR access to target sites in the genome is regulated and the mechanisms underlying subsequent GR-directed chromatin remodeling. We have now identified a novel regulator of this process, Merm1, that affects both GR access to target sites and also mediates GR-regulated histone modification. As we find that Merm1 protein is targeted for ubiquitin-mediated degradation by proinflammatory cytokine signaling, these discoveries link inflammation with gating of GR access to the genome and tissue sensitivity to glucocorticoid.

We initially identified Merm1 in a co-activator screen (27, 30). The co-activation effect was found to be dependent on both the SAM and methyltransferase domains, although previous studies had identified Merm1 as mediating induction of the repressive chromatin mark H3K9me. Further evidence for a role of Merm1 in transcriptional regulation is provided by a recent proteomic analysis of steroid receptor transcription factor complexes in which Merm1 protein was identified (47).

Merm1 gene expression was near ubiquitous in human tissues but with particularly high levels in bronchial epithelium, prompting examination of Merm1 in lung pathologies. We also studied Merm1 expression in immune cell types in response to activation, as these are prominent targets for GC therapy. We found induction of Merm1 gene expression in both B and CD8+ T lymphocytes, again supporting a role for Merm1 in inflammatory signaling.

As Merm1 is a candidate putative histone methyltransferase (26), we anticipated a role in chromatin modification but initially used an unbiased proteomic approach to gain insights into function and regulation. These studies revealed that Merm1 was a likely target for ubiquitinylation, Merm1 bound chromatin component proteins, and Merm1 did not bind directly to GR. However, Merm1 did co-immunoprecipitate with GRIP1, a chromatin-modifying enzyme, and GR co-modulator protein (13, 36). Merm1 also bound a number of enzymes, including histone acetyltransferases, associated with transcriptionally active chromatin, and the histone-binding protein RBBP7, which interacts with histone H3 in an H3K4me3-dependent manner (33).

Merm1 modulated GR regulation of a number of endogenous target genes, both positively and negatively. The well characterized GR target gene GILZ was analyzed further. GILZ is a potent anti-inflammatory mediator, and its induction by GR plays an important role in the anti-inflammatory repertoire activated by glucocorticoid treatment (48). GR binding to the two well defined GREs (35) was demonstrated along with Merm1.

Loss of Merm1 had a dramatic effect on recruitment of GR to the two GREs, but there was little impact on histone acetylation (Fig. 5*C*). Therefore, we examined candidate histone methylation changes. H3K4me3 is a reliable mark for active promoters, and we revealed a striking induction at the two GILZ GREs in response to ligand activation of the GR. In contrast, we also saw a marked decrease in the H3K79me2 mark (Fig. 5*E*). H3K79me2 is only known to be catalyzed by DOT1L and is best-characterized as an elongation chromatin mark associated with transcriptionally active chromatin (25, 49). Recent studies have demonstrated at a pan-genomic level asynchronous histone modifications accompanying gene transcription, and so this may be an example of actively remodeling chromatin driven by binding of the GR (50).

Merm1 knockdown dramatically reduced basal H3K79me2, especially at GL-3, but had minimal effects on basal H3K4me3. However, knockdown of Merm1 effectively abolished the GR induction of the H3K4me3 signal. Taken together this suggests that Merm1 is required for the maintenance of open chromatin at the GILZ locus to facilitate GR loading on the response elements. The increase in H3K4 trimethylation with reduction in H3K79 dimethylation in response to GR activation by ligand suggests coordinated histone modification whereby one change influences another. Loss of Merm1 prevents both changes, suggesting several possibilities. It may be that loss of the H3K79me2 mark inhibited GR recruitment and thereby acquisition of the H3K4me3 mark either catalyzed directly by Merm1 or the activating MLL complex recruited to the GR (20). Alternatively, the driving event may be the gain of methylation marks on H3K4 as a result of GR activation of Merm1 and that this mark serves to recruit histone methyltransferases targeting H3K79. However, it is clear that loss of Merm1 acts to regulate GR recruitment, and the chromatin mark observed most clearly under basal conditions is the loss of H3K79me2. For this reason it seems more likely that Merm1 is required for maintenance of H3K79me2.

There are many examples of collaborative and antagonistic relationships between different histone marks. For example CARM1 and PRMT1 act to enhance histone arginine methylation in response to estrogen receptor activation, and a further enzyme, PADI4, also a target of estrogen receptor, reverses the change by converting methylated arginine to citrulline (51). Also, dimethylation of H3R2 is prevented by H3K4me3 and vice versa (20).

We identified in a model of acquired GC resistance (combined IFNγ and TNFα) (36) that Merm1 protein expression was significantly reduced in a ubiquitination-dependent manner both in cell lines and human lung explants. In both cases loss of Merm1 was accompanied by loss of GR recruitment to GREs.

Our data suggested that Merm1 may be particularly important in the regulation of normal lung epithelial function. Therefore, we profiled Merm1 protein expression in a human lung tissue array and found a marked loss both in inflammatory and neoplastic disease. As pulmonary neoplasia frequently develops on a tissue background of chronic inflammation, it is perhaps no surprise to find a similar pattern of Merm1 expression in both disease processes. Of course, the earlier discovery of Merm1 as a regulator of distant metastases is also relevant in the context of pulmonary neoplasia (26).

Nuclear receptors are proposed to be pioneer factors capable of inducing open chromatin to permit binding of secondary regulatory factors (52–54). However, more recently genome-wide analysis suggests that the majority of GR binding occurs to constitutively accessible sites (10, 55). Our data identify Merm1 as a novel chromatin-modifying enzyme implicated not only in regulating GR access to target sites but also in mediating GR chromatin remodeling activity. We show also that Merm1 is negatively regulated by proinflammatory cytokines, resulting in impaired GC target gene regulation. We propose, therefore, that inflammation re-programs chromatin, in part through suppression of Merm1, affecting GR access to target genes and limiting subsequent chromatin remodeling activity. The Merm1-dependent GR cistrome is likely to be important in understanding how best to target pulmonary inflammation. Targeting Merm1 expression or function may be a viable strategy for potentiating glucocorticoid related anti-inflammatory action in human pulmonary inflammatory disease or may inform the design of novel GR ligands capable of re-directing GR in a Merm1-deficient cellular environment.

## Supplementary Material

Supplemental Data

## Footnotes

6 The abbreviations used are:

GC: glucocorticoid
GR: glucocorticoid receptor
GRE: glucocorticoid response element
H3K9: histone H3 lysine 9
Luc: luciferase
Bicine: *N*,*N*-bis(2-hydroxyethyl)glycine
SAM: *S*-adenosyl-l-methionine
CCL2: chemokine ligand 2
*TEV*: tobacco etch virus
ANOVA: analysis of variance
DEX: dexamethasone.
