# Supplementary material for: The Methyltransferase WBSCR22/Merm1 Enhances Glucocorticoid Receptor Function and Is Regulated in Lung Inflammation and Cancer
Source: J Biol Chem. 2014 Jan 31;289(13):8931–46. doi: 10.1074/jbc.M113.540906 (PMC3979408; doi:10.1074/jbc.M113.540906)
Supplement: Supplemental Data [file supp_289_13_8931__index.html]

The Methyltransferase WBSCR22/Merm1 Enhances Glucocorticoid Receptor Function and is Regulated in Lung Inflammation and Cancer. — The Methyltransferase WBSCR22/Merm1 Enhances Glucocorticoid Receptor Function and Is Regulated in Lung Inflammation and Cancer — Merm1 Mediates Glucocorticoid Resistance — Supplemental Data 

# The Methyltransferase WBSCR22/Merm1 Enhances Glucocorticoid Receptor Function and Is Regulated in Lung Inflammation and Cancer

## Supplemental Data

**Files in this Data Supplement:**

- Supplemental Data (.pdf, 359 KB) - Supplemental Methods and results
- Supplemental Data (.xlsx, 46 KB) - List of Merm1 and pHalo interacting proteins
