# Supplementary material for: The Methyltransferase WBSCR22/Merm1 Enhances Glucocorticoid Receptor Function and Is Regulated in Lung Inflammation and Cancer
Source: J Biol Chem. 2014 Jan 31;289(13):8931–46. doi: 10.1074/jbc.M113.540906 (PMC3979408; doi:10.1074/jbc.M113.540906)
Supplement: Supplemental Data [file supp_M113.540906_jbc.M113.540906-1.pdf]

Supplemental Information

Supplementary materials and methods

ChIP primers

GILZ1 Fwd AGT TGG TAC AAG AAA GTG C
Rev CTC GTA TGT CAC AAA CTC C; GILZ2 Fwd AAA GAG TAG AAT GTG GAG ACT Rev GCA CAG GTC CAT GCT AAT C; GILZ3 Fwd GGG AAT TCT GAT ACC AGT TAA GC, Rev GGG AGA CAA TAA TGA TCT CAG GA.

Steroid co-regulator primers

SRC1 Fwd TGCCTC CGG GTA TCA GTC ACC AG, SRC1 Rev AGG CGT GGG CTG GTT CTG GAC AG; GRIP1 Fwd GTG GTA TGC CAG CAA CTA TGAGC,
GRIP1 Rev TGG ATC AGG TTG CTGACT TAT TCC G; CARM1Fwd CACACCGACTTCAAGGACAA ,CARM1 Rev AAAAACGACAGGATCCCAGA. B-Actin Fwd GTG GGG CGC CCC AGG CAC CA, B-Actin B Rev CTC CTT AAT GTC ACG CAC GAT TTC.

Merm1 mutagenesis

Mutagenesis was carried out using Quik Change kit, used according to the manufacturer's instructions (Agilent Technologies) using the following primers: Merm1 K180RFwd GATCACAACCCA GGCCACAAGGGCAGG CTTCTCCGGTGG, Merm1 K180RRev CCACCGGAGAA GCCTGCCCTTGTGGC CTGGGTTGTGATC,
Merm1 K196RFwd GACTACCCT AACAGTGCCAGAGCAAAG AAATTCTACCTC Merm1K196RRev GAGGTAGAAT TTCTTTGCTCTGGCA CTGTTAGGGTAGTC.

Supplementary figure legends

Figure S1. Merm1 overexpression does not enhance trans-repression. HeLa cells were transiently co-transfected with NRE-Luc, and as indicated with either GRIP1, Merm1, or an empty plasmid (pcDNA3 or cmv.SP6 vector see experimental procedures section for details), and treated with 0.5 or 5 ng/ml TNFα and 100 nM Dex for 16 hours before luciferase assay. Results are expressed as fold repression (mean and standard deviation), experiments were performed in triplicates, on three separate occasions

Figure S2. Merm1 co-activates MR, PR and AR. HEK293T cells were transiently co-transfected with 0.5μg of TAT3-Luc reporter, 0.01μg Renilla reporter, 0.3 μg of Merm1 or cmv.SP6 empty vector (EV) and 0.5 μg of Mineralocorticoid receptor (MR), progesterone receptor (PR) or androgen receptor (AR) expression vectors, as indicated. Twenty four hours after transfection, cells were incubated with corticosterone (10nM) for MR; progesterone (10nM) for PR; or dihydrotesterone (DHT) (10nM) for AR, for 16hrs before luciferase assay. Graphs show mean and standard deviation, experiments were performed in triplicates, on three separate occasions. RLU, corrected relative light unit. ** $p<0.01$ data compared using independent samples Student's t test.

Figure S3. Expression of Merm1 mutants in HEK293T cells. Merm1 mutants were expressed in HEK293T cells (as described in the experimental procedures). Expression levels were determined by western blotting, using an anti-Merm1 or β-actin antibody to determine equal loading (molecular weight markers shown in kDa). Control Sp6 (empty plasmid), Merm1 wild type [WT] (32 kDa), Merm1 nuclear localisation domain mutant, ΔNL (29 kDa); Merm1 SAM-domain mutant, ΔSAM (14 kDa), and Merm1 methyltransferase domain mutant, ΔMethT (29 kDa).

Figure S4. Merm1 siRNA does not affect other steroid co-regulators. A549 cells were transfected with 10nM (s41530) or control siRNA (Dharmacon siCONTROL Nontargeting siRNA) for 48 hours and prior to RNA extraction. The expression of Merm1, SRC1, GRIP1 and CARM1 were measured by qRT-PCR, with β-actin as a house-keeping gene. Graphs show mean gene expression and standard deviation, experiments were performed in triplicates, on three separate occasions.

Figure S5. Merm1 siRNA inhibits DEX-induced gene expression. A549cells were transfected with 10nM Merm1(s41530) or control siRNA (Dharmacon siCONTROL Nontargeting siRNA) for 48 hours. Cells were then treated with dexamethasone (DEX 100 nM) for 2 hours and then RNA extracted. The expression of the GILZ gene was measured by qRT-PCR, with β-actin as a house-keeping gene. Graphs show mean gene expression and standard deviation, experiments were performed in triplicates, on three separate occasions. * $p<0.05$ data compared using independent samples Student's t test.

Figure S6. Merm1 siRNA inhibits DEX-induced MMTV-reporter gene activity. A549 cells were transfected with 10nM Merm1 or control siRNA for 24 hours and then were transiently co-transfected with 0.5μg of MMTV-Luciferase reporter, 0.01μg Renilla reporter and incubated for a further 24 hours, cells were then treated with DEX (100 nM), for 16hrs before luciferase assay. Graphs show mean luciferase and standard deviation, experiments were performed in triplicates, on three separate occasions * $p<0.05$ data compared using independent samples Student's t test.

Figure S7. GILZ1 region (GL-1) serves as a non-GR binding (negative) control region for the ChIP assay. HeLa cells were treated with vehicle or 100nM Dex for 1hr. Following fixation and chromatin extraction (see Experimental procedures), ChIP assays were

performed using antibodies against, GR and Merm1 (with IgG as a control). GR binding regions were analysed with primer pairs for GL-1, 2 and 3 promoter regions. PCR products were resolved on agarose gels and visualized by ethidium bromide staining (representative images shown, 3 individual gels).

Fig S1

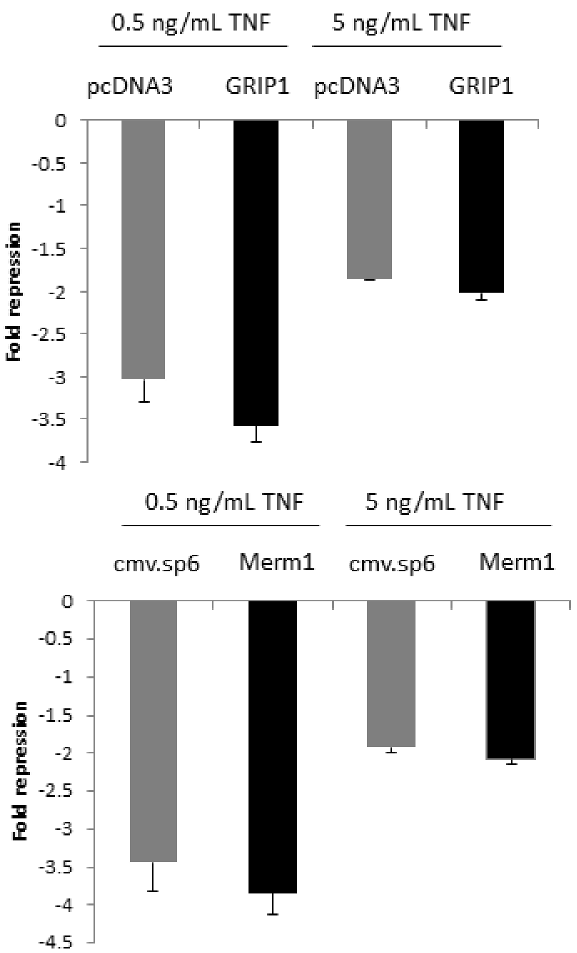
0.5 ng/mL TNF
5 ng/mL TNF
pcDNA3
GRIP1
pcDNA3
GRIP1
Fold repression
0
-0.5
-1
-1.5
-2
-2.5
-3
-3.5
-4
0.5 ng/mL TNF
5 ng/mL TNF
cmv.sp6
Merm1
cmv.sp6
Merm1
Fold repression
0
-0.5
-1
-1.5
-2
-2.5
-3
-3.5
-4
-4.5

Fig S2

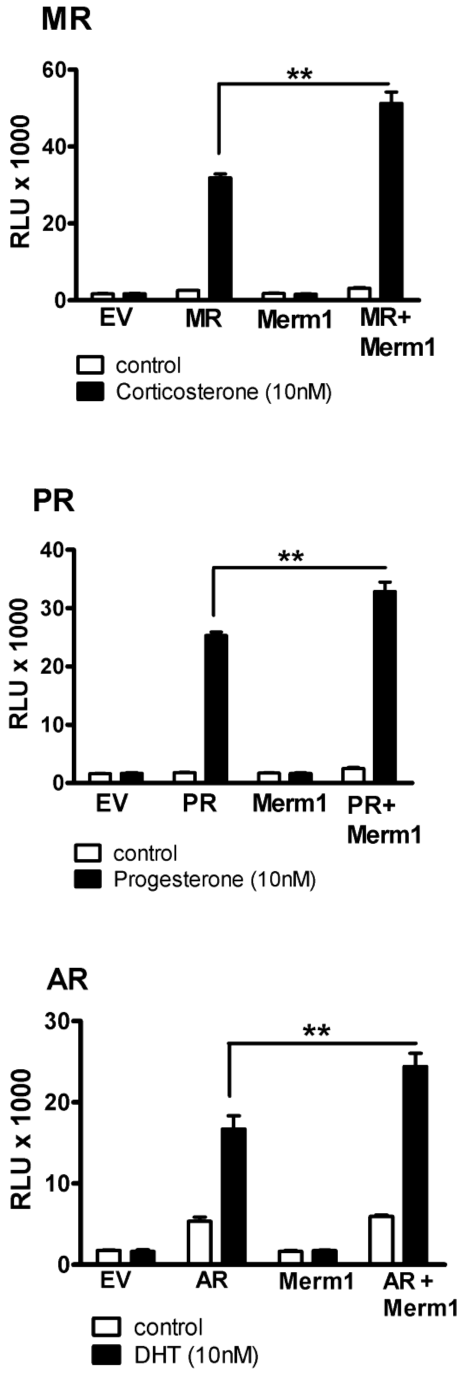
MR
RLU x 1000
60
40
20
0
**
EV
MR
Merm1
MR+
Merm1
control
Corticosterone (10nM)
PR
RLU x 1000
40
30
20
10
0
**
EV
PR
Merm1
PR+
Merm1
control
Progesterone (10nM)
AR
RLU x 1000
30
20
10
0
**
EV
AR
Merm1
AR +
Merm1
control
DHT (10nM)

Fig S3

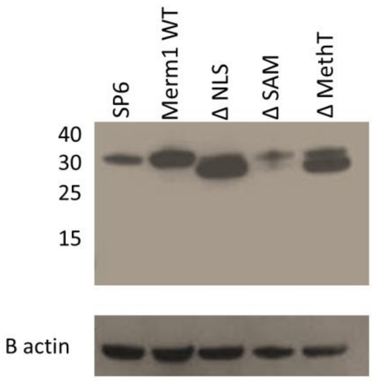

Fig S4

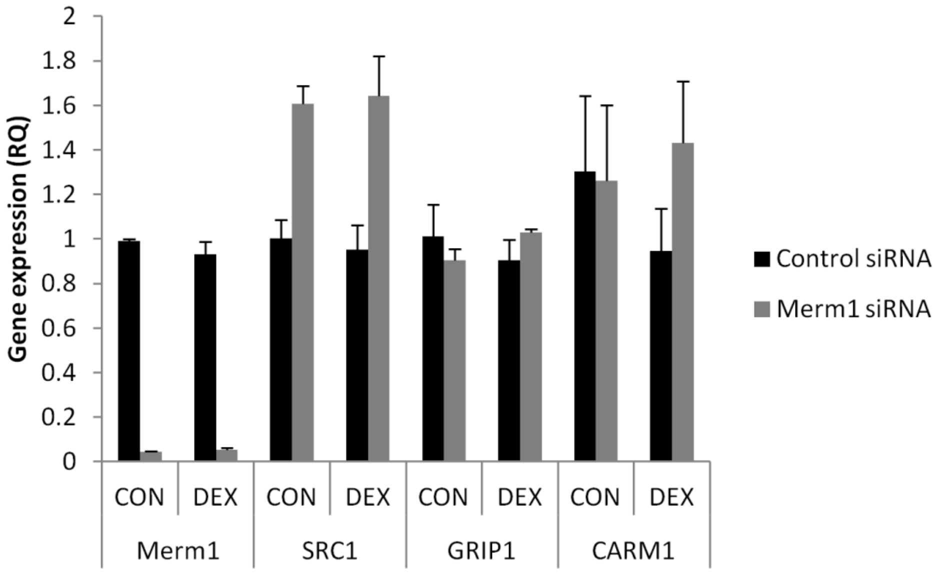

Gene expression (RQ)
2
1.8
1.6
1.4
1.2
1
0.8
0.6
0.4
0.2
0
CON
DEX
CON
DEX
CON
DEX
CON
DEX
Merm1
SRC1
GRIP1
CARM1
Control siRNA
Merm1 siRNA

Fig S5

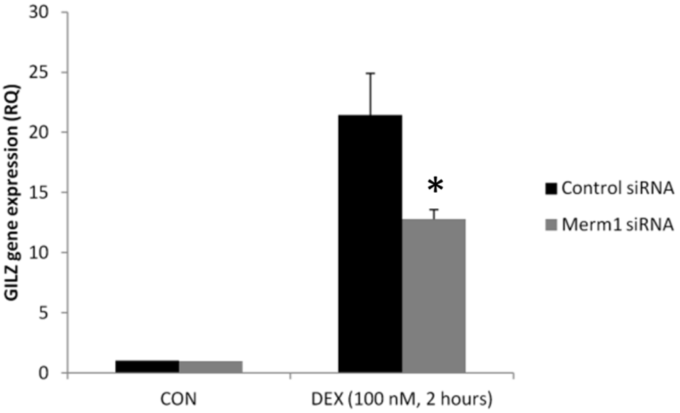
30
25
20
15
10
5
0
GILZ gene expression (RQ)
*
Control siRNA
Merm1 siRNA
CON
DEX (100 nM, 2 hours)

Fig S6

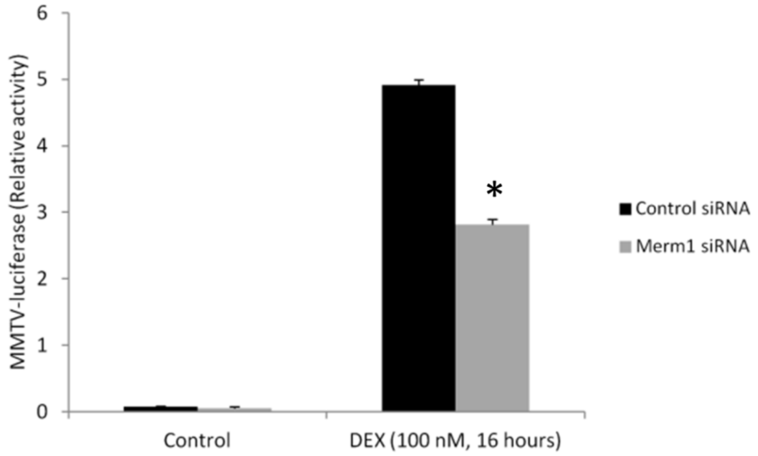
MMTV-luciferase (Relative activity)
6
5
4
3
2
1
0
*
Control
DEX (100 nM, 16 hours)
Control siRNA
Merm1 siRNA

Fig S7

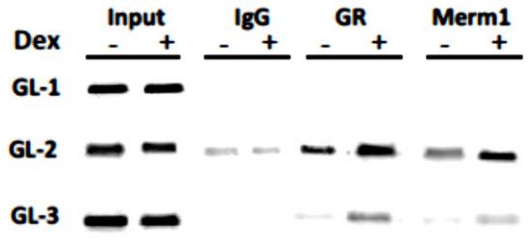

Supplementary table 1 Data-Merm1 interacting proteins and network analysis. Full list of interacting proteins is available (see merm1 and pHalo interacting proteins.xls)

<u>Table S1- Merm1 interacting proteins</u>

**Merm1 interacting protein**	**Peptides and %Coverage**	**Function (taken from Uniprot)**
RuvB-like 1 and 2	7/41.9 and 5/28.3	A member the NuA4 histone acetyltransferase complex
E3 ubiquitin-protein ligase HUWE1	11/13.9	E3 ubiquitin-protein ligase that mediates ubiquitination and proteasomal degradation
E3 ubiquitin-protein ligase UBR5	15/23.0	E3 ubiquitin-protein ligase and regulator of DNA damage responses
Nucleosome assembly protein 1-like 1 (NAP1)	5/50.24	Interacts with histones, possibly involved with chromatin formation and cell proliferation
HCLS1-associated protein X-1 (HAX1)	5/53.7	Promotes cell survival and cell migration, interacts with XIAP, cleaved by caspase-3 during apoptosis
Nuclease-sensitive element-binding protein 1	3/53.7	Involved in pre-mRNA splicing regulation. Binds and stabilizes cytoplasmic mRNA. Component of the CRD-mediated complex that promotes MYC mRNA stability.
Histone-binding protein RBBP7	4/35.4	Core histone binding protein, targets chromatin remodeling factors
Histone acetyltransferase type B (HAT1)	4/29.4	Acetylates soluble but not nucleosomal histone H4. Possibly involved in nucleosome assembly during DNA replication and repair.
Proteasome activator complex subunit 3	2/22.8	Subunit of the 11S REG-gamma proteasome regulator.
v-Raf-1 murine leukemia viral oncogene homolog 1 (RAF1)	1/13.5	Serine/threonine-protein kinase, regulation of mitogen-activated protein kinase cascades
Melanoma-associated antigen D2	10/36.3	Suppresses the expression of tumor necrosis factor-related apoptosis-inducing ligand (TRAIL) death receptor 2
Cyclin-dependent kinase inhibitor 2A	1/6.1	Tumour suppressor, interacts with UBE2I/UBC9 and enhances sumoylation of a number of its binding partners, also binds to HUWE1

Cyclin-dependent kinase 1	3/40.71	serine/threonine kinase, cell cycle control
tRNA methyltransferase 112 homolog (Trmt112)	4/53.6	Participates both in methylation of protein and tRNA species. The heterodimer with HEMK2/N6AMT1 catalyzes N5-methylation of ETF1 on 'Gln-185', using S-adenosyl L-methionine as methyl donor.
rRNA 2'-O-methyltransferase fibrillarin (FBL)	4/50.5	Involved in pre-rRNA processing. Utilizes the methyl donor S-adenosyl-L-methionine to catalyze the site-specific 2'-hydroxyl methylation of ribose moieties in pre-ribosomal RNA
RNA methyltransferase-like protein 1 (RNMT1)	1/11.9	
RNA-dependent helicase p72 (DDX17)	1/7.3	RNA-dependent ATPase activity. Involved in transcriptional regulation. Transcriptional coactivator for estrogen receptor ESR1
